# Negative Emotional Stimuli Enhance Conflict Resolution Without Altering Arousal

**DOI:** 10.3389/fnhum.2019.00282

**Published:** 2019-08-13

**Authors:** Daniel J. Fehring, Ranshikha Samandra, Marcello G. Rosa, Farshad A. Mansouri

**Affiliations:** ^1^Cognitive Neuroscience Laboratory, Department of Physiology, Monash Biomedicine Discovery Institute, Monash University, Clayton, VIC, Australia; ^2^ARC Centre of Excellence for Integrative Brain Function, Monash University, Clayton, VIC, Australia; ^3^Department of Physiology, Monash Biomedicine Discovery Institute, Monash University, VIC, Australia

**Keywords:** emotional modulation of cognitive functions, executive control, conflict-induced behavioral adjustment, arousal, Wisconsin Card Sorting Test

## Abstract

In our daily life, we frequently need to make decisions between competing behavioral options while we are exposed to various contextual factors containing emotional/social information. We examined how changes in emotional/arousal state influence resolving conflict between behavioral rules. Visual stimuli with emotional content (positive, negative and neutral) and music (High/Low tempo), which could potentially alter emotional/arousal states, were included in the task context while participants performed the Wisconsin Card Sorting Test (WCST). The WCST requires the application of abstract matching rules, to resolve conflict between competing behavioral options. We found that conflict influenced both accuracy and response time (RT) in implementing rules. Measuring event-related autonomic responses indicated that these behavioral effects were accompanied by concomitant alterations in arousal levels. Performance in the WCST was modulated by the emotional content of visual stimuli and appeared as a faster response and higher accuracy when trials commenced with negative emotional stimuli. These effects were dependent on the level of conflict but were not accompanied by changes in arousal levels. Here, we report that visual stimuli with emotional content influence conflict processing without trial-by-trial changes in arousal level. Our findings indicate intricate interactions between emotional context and various aspects of executive control such as conflict resolution and suggest that these interactions are not necessarily mediated through alterations in arousal level.

## Introduction

Executive control (Miller and Cohen, 2001; Mansouri et al., 2016b) is essential for optimizing the flexible use of limited cognitive resources to currently prioritized tasks, in order to support goal directed behavior (Mansouri et al., 2009, 2015b, 2016b; Bonte et al., 2011). This control, amongst other processes, may be achieved via the detection and resolution of conflict between behavioral choices (Botvinick et al., 2001; Mansouri et al., 2007, 2009; Marchewka et al., 2014; van Steenbergen et al., 2009; Olk et al., 2015). Conflict-induced behavioral adjustments appear as a decline in performance when conflict arises in a trial (conflict cost) and also as an improved ability to resolve the conflict in the subsequent trial (conflict adaptation) (Mansouri et al., 2009, 2017b).

Recent studies indicate that executive control processes are influenced by emotional factors (van Steenbergen et al., 2009, 2010; Inzlicht et al., 2015; Saunders et al., 2015). There has been long lasting debate regarding the mechanisms underlying the interaction of emotion and executive control of behavior when a choice between conflicting-competing options becomes necessary (van Steenbergen et al., 2009, 2010; Fritz and Dreisbach, 2015). The dual competition model explains the interaction of emotion and executive functions in terms of shared processing resources (Egner et al., 2008; Mansouri et al., 2009; Pessoa, 2009; Etkin et al., 2011; Padmala et al., 2011). This model postulates that task performance is impaired in the presence of any task-irrelevant stimuli, whether emotionally salient or not, as resources for the primary task are utilized toward processing this external stimuli (Padmala et al., 2011). This proposition was validated in a study which revealed that the presentation of task-irrelevant images presented immediately prior to trials, increased reaction time (Hart et al., 2010; Padmala et al., 2011). Other studies also suggest that the interaction between executive functions and emotional regulation (Garcia et al., 2011) might be mediated through overlapping neural substrates as imaging studies have revealed a large overlap in brain areas which support executive functions and those which regulate emotional state (Bock, 2010). In this view, the demand for attentional-cognitive resources by each regulatory system might shape the outcome of interaction between emotional stimuli and executive functions and influence the ability to resolve conflict in various cognitive tasks.

In contrast to this, other models propose that priming of emotional state may specifically influence conflict monitoring and resolution (van Steenbergen et al., 2009, 2010). This model stems from the proposal that conflict is innately aversive (Botvinick, 2007; Padmala et al., 2011), and postulates that conflict resolution is sensitive to emotional state, as it is dependent on the assessment of conflict as an aversive cue (van Steenbergen et al., 2010). This proposal was validated in studies where conflict-induced behavioral adjustments were either attenuated when paired with a reward, as the reward diminished the aversive assessment of the conflict (van Steenbergen et al., 2010), or enhanced when paired with a negatively valenced stimuli, as the stimuli heightened the aversive assessment of the conflict (Padmala et al., 2011).

Other models also propose that conflict has an affective cost (a negative influence upon emotion) while inducing behavioral adaptation to reduce further costs if this conflict reappears (van Steenbergen and Band, 2013). A related view proposes that resolving conflict is cognitively demanding and has an inherent cost, thus people aim to minimize cognitive efforts and avoid instances which induce them (van Steenbergen and Band, 2013). Complimentary to this, it has been shown that conflict between behavioral choices trigger negative emotional state and avoidance behavior (Schouppe et al., 2012; van Steenbergen and Band, 2013). It has been proposed that such negative emotion is an important factor in triggering and recruiting executive control to resolve the conflict (Inzlicht et al., 2015). These studies suggest that the emergence of conflict and the efforts to resolve it or avoid its reoccurrence are associated with emotional state change and therefore what appears as interaction of emotion and executive control is an inherent property of conflict processing (Inzlicht et al., 2015).

The interaction of emotional state and conflict might also be considered as a subset of a broader model of interaction between arousal-emotional state and decision-making processes. The somatic marker hypothesis postulates that alterations in emotional regulation and arousal state may influence the decision-making process (Bechara et al., 1999, 2005). In this context, conflict and its associated cognitive difficulty or uncertainty might induce emotional state change with concomitant autonomic and somatic responses and consequently influence executive functions and conflict resolution (Mansouri et al., 2017a).

In this study, we aimed at examining the interaction of background emotional information on executive functions, and specifically conflict processing. A prominently used neuropsychological test for assessing executive control, particularly the ability in resolving conflict between competing behavioral rules, is the Wisconsin Card Sorting Test (WCST) (Berg, 1948; Stuss et al., 2000; Mansouri et al., 2016a). The WCST assesses the participants’ ability to shift between abstract rules (which change without notice) using trial and error (Mansouri et al., 2016a). Previous studies have revealed that this rule-shifting leads to conflict between potential rules, and impairs both accuracy and response time (RT) (conflict cost) (Monsell, 2003; Aron et al., 2004; Mansouri et al., 2016a).

We hypothesized that contextual factors with emotional content might convey emotional information and influence cognitive functions, particularly when resolving conflict requires recruitment and allocation of executive control (Mansouri et al., 2017a). Such an interaction between emotional regulation and cognitive control might influence the ability to resolve the conflict. Therefore, we introduced two sources (from two different modalities) of information with emotional content, “visual stimuli with emotional/social content” and “music,” while participants performed different versions of the WCST.

Previous studies have revealed that music may improve (Salamé and Baddeley, 1989; Bottiroli et al., 2014) or reduce (Furnham and Strbac, 2002; Brown et al., 2006) performance in various perceptual, motor, and cognitive tasks (Mansouri et al., 2016a, 2017a; Feizpour et al., 2018). It has been proposed that these effects may be mediated through the direct influence of music over executive functions by limiting available cognitive resources (Koelsch et al., 2006; Gyurak et al., 2011; Moore, 2013). Music may also influence emotional state and arousal (Cockerton et al., 1997) and indirectly alter the interaction of emotional and executive control processes, consequently modulating performance in cognitive tasks (Haddon and Killcross, 2006; Mansouri et al., 2016a). Our previous studies have indicated that music tempo is a crucial component of the acoustic stimuli which alters subjective experience, so that high- and low-tempo music evoke happy and sad feelings, respectively (Supplementary Material Control Study 2). In addition, high-tempo, but not low-tempo music, significantly affected the practice-related alterations in the response inhibition and this was accompanied by concomitant changes in arousal response in the context of Stop signal task (Mansouri et al., 2017a). Therefore, in this study we included high-tempo and low-tempo music as background acoustic conditions while participants performed the cognitive task and hypothesized that music would affect the emotional-arousal state and influence conflict resolution.

Previous studies have also shown that visual stimuli with emotional content influence performance in the context of various cognitive tasks (McKenna and Sharma, 1995; Tipples and Sharma, 2000; Rudebeck et al., 2006; Zarei et al., 2019). Inclusion of two sources of emotional stimuli from two different modalities (visual stimuli and music) could potentially help delineating the interaction of various emotional stimuli in influencing conflict processing. If both emotional factors elevate the arousal level their additive effects might appear as larger alterations in behavioral measures in the cognitive task.

We also monitored participant’s event-related arousal/emotional response during cognitive task performance in all conditions. Rapid transient shifts in electrodermal activity (EDA) (Wallin, 1981; Critchley, 2002; Lang and Bradley, 2010) can be measured as an index of arousal level and reflect emotional state change by aspects of cognitive task (e.g., conflict and error commission) and/or external modulating factors, such as music (Zimny and Weidenfeller, 1963; Bechara et al., 1999; Khalfa et al., 2002; Hajcak et al., 2003; Garcia et al., 2011; Ullsperger et al., 2014; Ebitz and Platt, 2015; Mansouri et al., 2017a). We hypothesized that if changes in arousal level mediate the interaction of emotion and conflict processing, this would be reflected in concomitant changes in event-related EDA.

## Materials and Methods

### Participants

Fifty-five Monash University undergraduate (third year) students (35 female) were recruited to complete the WCST in three separate 2-hour sessions, each one week apart. *A priori* power analysis was performed for the estimate of required sample size based on data obtained and reported in our recently published study (Mansouri et al., 2016b). The effect size for conflict-induced behavioral adjustment (conflict adaptation) was estimated using Cohen’s criteria (Cohen, 1988) based on $\eta_{p}^{2}$, which was 0.156. With an alpha set at 0.05, and power set at 0.80, the estimated sample size required for this effect size was 36 participants (using G^*^Power 3.1 Faul et al., 2007). To achieve counterbalancing for all conditions we recruited even more participants. Thus, our sample size of 55 is adequate for the detection of the smallest effect with 80% power, given the current design. All participants (age range between 20 and 27) had similar educational background and no history of any neurological disorders. Approval was obtained from Monash University Human Research Ethics Committee. Written and informed consent was obtained from all participants.

### Apparatus

An automated test apparatus was used to perform the WCST. Participants were seated in front of a switch and a touch-sensitive screen (17^″^ MicroTouch surface capacitive touch display) on which the stimuli were displayed. Participants were approximately 60 cm from the screen and were informed to maintain focus in the middle of the screen. The size of each stimulus ranged between 5 and 7 cm. The switch was fixed on a pad with a wrist rest in line with the bottom center of the screen. The subjects were informed to only use their dominant index finger to press the switch and then the target stimulus on the screen.

Participants were also monitored via camera during the completion of the task. Data acquisition was controlled by CORTEX program (National Institute of Mental Health) at millisecond resolution. Before performing the WCST, participants read an explanatory note regarding the test procedure following this a structured verbal briefing was also provided, using a written script to ensure consistency.

### Procedure

**FIGURE 1:**
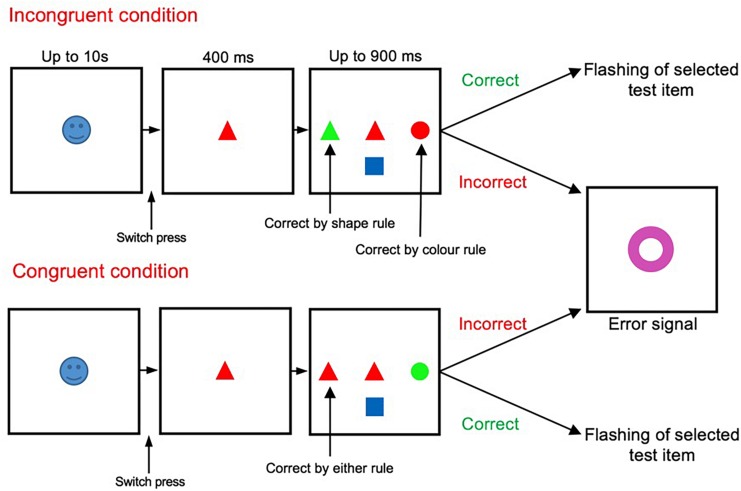
Wisconsin Card Sorting Test (WCST). The incongruent (also termed high-conflict) and congruent (also termed low-conflict) trials were randomly presented, in equal proportion, throughout the testing period.

The main task shown in Figure 1 has been described and validated in previous studies (Mansouri et al., 2007, 2014, 2016a). The start cue was an emotionally salient image (positive, negative or neutral images) presented randomly at the start of each trial. After the start cue onset, participants had to press the switch with their dominant index finger within 10 s. If the switch was pressed, the start cue was replaced by a sample stimulus (for 400 ms). If the participant kept the switch pressed, three test items appeared surrounding the sample (to the left, right, and below the sample stimulus). The sample stimuli presented were selected at random from a set of 36 stimuli (combination of 6 colors and 6 shapes). The test items were also selected from this pool at random (with restrictions imposed to meet the necessity to generate either a congruent or incongruent condition). Once test items were displayed, participants had to rapidly release the switch and touch the appropriate test item that matched the sample according to the relevant rule. Participants had to respond as quickly as possible within a limited time window (900 ms). Failure to touch the screen within this window was considered as a time-out error. If target selection was correct, confirmation feedback was given to the subject (the target item flashed twice). However, if erroneous, all presented stimuli were replaced with a large error signal (a purple annulus) which was displayed for 500 ms. Early release of the switch during the presentation of the sample stimuli alone was also considered as an error.

The appropriate matching rule (either color or shape) was maintained in blocks of trials, and changed without any notice when the shift criterion (9 correct responses from 10 trials) was met. Within the task there were two trial types, incongruent and congruent, with two levels of conflict between the behavioral rules (Figure 1). Within incongruent trials the sample stimuli matched one of the test items in color, yet another item in shape. The other test item did not match the sample in either shape or color. Therefore, participants had to resolve the conflict between the two potentially matching targets in applying the appropriate rule and selecting the correct target item. However, within congruent trials, the sample stimuli matched one of the test items in both color and shape, but did not match with the other test items in color nor shape. Thus, in congruent trials there was only one potentially matching target.

Each weekly testing session comprised of two stages of the WCST with a 10-minute break between stages to minimize fatigue effects. Each of these two testing stages ran for approximately 30 to 45 minutes, dependent on performance. The task commenced with three practice blocks, in which data was not analyzed. The first practice block included only congruent trials, while the second and third practice block included only incongruent trials, requiring the application of the color or shape rule, respectively. Within these practice blocks the shift criterion was set at 5 correct responses from 5 trials. Following these practice blocks, 10 blocks were ran, alternating between the color or shape rule with the aforementioned rule shift criterion (9 correct responses from 10 trials).

### Electrodermal Activity Recording

Event-related EDA was recorded, in microsiemens (μS), continuously for the entire duration of the task (at 75 kHz sampling rate using an electrodermal recording unit: ML116 GSR Amp and PowerLab 26T, ADInstruments). Task-relevant events were simultaneously encoded during EDA recording. The electrodes were connected to the palmar surface of the index and ring fingers of the non-dominant hand, and participants were informed to keep this hand stationary during testing.

Amplitude of phasic activity was measured as the difference between the maximum and minimum value of the EDA waveform within an event-related epoch (Wolfram, 2012). Within this study, two epochs were used: a post-feedback epoch [response selection onward (4 s)], to examine changes in arousal level after the awareness of trial outcome, and a pre-feedback epoch [From the start cue to response feedback onset (2.4 s window)], to examine changes in arousal level before feedback.

### Visual Images With Emotional Content

We used music and visual stimuli as “background contextual stimuli” without any relation to the rule-based target selection which was the main task in the WCST. For visual stimuli, multi-color images of items and faces were categorized (by one female and one male) to images with positive, negative and neutral emotional content.

At the start of each trial, multi-colored natural or cartoon images including human faces were shown as the Start-cue. These visual images were categorized to three conditions with positive, negative and neutral emotional content by one female and one male before the start of data collection. Visual images were not repeated in the testing session to avoid multiple exposure (trial unique design). Participants performed the test in three consecutive weeks and none of the visual stimuli was repeated. Trial unique design for exposing the participants to emotional stimuli was a crucial aspect of our study because any repeated exposure would have introduced confounding issues such as familiarity and memory processing and indirectly evoked other cognitive processes and associated changes in arousal state. Participants were not aware of the categorization of the visual images based on their emotional content and were instructed to press a switch and initiate the trial as soon as they see any image. The presentation of the images as start cues, enables the examination of the influence of emotional stimuli on behavior and on arousal state. Concomitant alterations in arousal level was assessed in before (pre-feedback epoch) or after feedback (post-feedback epoch) to participants’ responses.

We also replicated the study using stimuli from the Nencki Affective Picture System (NAPS). NAPS is a large collection of multicolor photographs classified into five categories (People, Faces, Animals, Objects, and Landscapes) and are controlled in terms of “dimension,” “luminance,” “entropy,” and “contrast“(Marchewka et al., 2014; Riegel et al., 2016). In Supplementary Material Control Study 2 a subset of 360 images (same size of image pool as the main study) were randomly selected from the NAPS pool of images (120 for each emotional category: positive, negative, neutral). In NAPS, the emotional aspects of images were rated on a scale of 1–9 by 204 people, on subjective “valence” and “arousal.” We assigned images to three separate categories (Positive, Negative, and Neutral) based on each image’s ranking for Affective valence (Marchewka et al., 2014). Images within a range of 1.00–2.50, 3.75–6.25, and 7.50–9.00 were used for the selection of Negative, Neutral, and Positive image pool, respectively.

### Music

In each of the three weekly testing sessions, participants listened to one of the three types of music: high tempo with lyrics, low tempo with lyrics, or no-lyric music (of mixed tempo) for the duration of the task. We included no-lyric music to control for any effect of lyrics. We first selected a set of pop songs and then classified them based on their tempo: low [80–100 beats per minute (bpm)] and high tempo (120–140 bpm) music. Songs were excluded if they contained any offensive lyrical statements. The volume of the music across sessions was set (at 70 decibels), however, participants were able to change it only if it was too loud or quiet. The order of the three music conditions (low tempo, high tempo and no-lyrics) was counterbalanced across weekly testing sessions (see Supplementary Table S1 for the list of songs).

### Data Analyses

Each of the daily testing sessions comprised of two testing stages (first and second), which allowed assessment of within-session practice-related learning (Fehring et al., 2019). We measured the time from the onset of the target stimuli to the first touch on the screen as RT. Repeated-measure Analysis of Variance tests (ANOVA) were used to assess the effects of practice, emotional images and music on various behavioral measures. All data points were used in data analyses without removal of outliers. In this study, participants had to deliver a response within a limited response window (900 ms) and if participants could not deliver their response within this window the trial was assigned as a timeout trial. Therefore, all RT data used for analyses were within this response window. Implementing arbitrary procedures for the removal of outliers might bias the results and outcome of statistical analyses. Therefore, we followed our previously published approach (Mansouri et al., 2016b, 2017a) and did not remove any data point as outlier and included all data points in the statistical analyses. Distributional properties for key measures are shown in Supplementary Figure S2.

To ease comparison of the emotional conditions (such as positive, negative, neutral) and different sessions, RT and EDA were calculated for each condition in each testing stage and then normalized by dividing each value by the grand average for all conditions. We have implemented such normalization procedure for RT (Mansouri et al., 2007, 2015a, 2016b, 2017a) and EDA (Mansouri et al., 2017a) in our previous studies. Early switch release during sample presentation was considered as an error and participants received an error signal to avoid such errors, however, these procedural errors were not included in the calculation of accuracy. When color- or shape-matching was required, the majority of errors were perseverative errors (selecting the target based on irrelevant rule) or timeout trials and participants rarely committed non-perseverative errors (selecting the target that did not match the sample by either color or shape). Accuracy was calculated as the percentage of correct trials [correct trials/(correct + perseverative errors + non-perseverative error + timeout trials)] and analyzed without normalization. All participants successfully completed the required number of rule-shifts and achieved high percentage of correct responses in the WCST and therefore accuracy data were used without normalization (Mansouri et al., 2015a, 2016a, 2017a).

Each participant performed rule-based target selection at different conflict levels and therefore Conflict was included in a ANOVA as a within-subject factor. Each participant was exposed to all categories of visual images and different background Music conditions. Therefore, Emotion (visual stimuli with emotional content: positive/negative/neutral) and Music (high-tempo/Low-tempo/mixed tempo with no-lyrics) were included in the ANOVA as within-subject factors. For repeated-measure ANOVA, sphericity was examined (Mauchly’s test) and Greenhouse-Geisser correction was applied when necessary. The alpha level was set at 0.05 for all statistical tests. For significant effects, $\eta_{p}^{2}$ was also reported, which indicates the proportion of the variance explained by the effect in ANOVA analysis. Where significant interactions were detected pairwise comparisons were conducted. All pairwise comparisons were two-tailed *t* test with Bonferroni adjustment for multiple comparison. To ease interpretation when presented graphically: Significance level of ^*^p < 0.05, ^∗∗^*p* < 0.01, and ^∗∗∗^*p* < 0.001. No additional manipulations of data or measures were implemented.

## Results

We first report the results obtained in the Main blocks in which color or shape was the relevant matching rule. Participants rarely (<10%) chose the test item which did not match the current rule (color or shape), and only rarely committed errors in congruent trials. Within incongruent trials, most errors committed were perseverative, defined as selecting the target item which matched the sample by the alternative rule. These perseverative errors were mostly committed immediately after the rule change.

### Emotionally Negative Visual Stimuli Enhance Conflict Resolution

Music and visual stimuli with emotional content can potentially influence the emotional/arousal state. To examine the interaction of these factors with aspects of executive control such as conflict processing and practice-related learning, we applied a multi-factor ANOVA [Conflict (congruent/incongruent trials, within-subject factor) × Music (high tempo with lyrics/low tempo with lyrics/mixed tempo with no-lyrics, within-subject factor) × Practice (first/second stage of testing, within-subject factor) × Emotion (visual stimuli with emotional content: positive/negative/neutral, within-subject factor)] to RT in correct trials. The main effect of Conflict was significant, *F*(1,54) = 222.09; *p* < 0.001, $\eta_{p}^{2}$ = 0.80, indicating that the higher level of conflict in incongruent trials increased RT (conflict cost) (Figure 5A). There was a significant main effect of Practice, *F*(1,54) = 14.51; *p* < 0.001, $\eta_{p}^{2}$ = 0.21, indicating that RT decreased from the first to the second stage in the same testing day (within-session learning).

**FIGURE 2:**
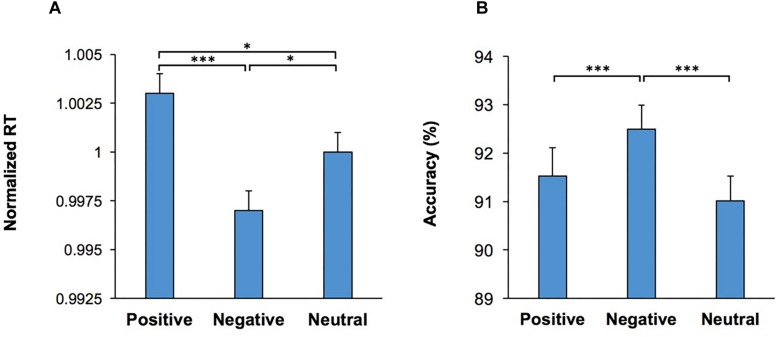
Emotional content of images shown as the start cue influenced performance in the WCST. **(A)** Response time (RT) in correct trials are shown for each emotional category. RT was lowest in trials which contained a negative visual stimulus (shown as the start cue) **(B)** The percentage of correct trials are shown for each emotional category. Accuracy was the highest in trials which contained a negative visual stimulus. ^*^represents *p* < 0.05, ^∗∗∗^represents *p* < 0.001.

Importantly, the main effect of Emotion was significant, *F*(2,108) = 9.20; *p* < 0.001, $\eta_{p}^{2}$ = 0.15, indicating that the emotional content of the visual stimulus influenced RT. Pairwise comparisons (two-tailed *t* test with Bonferroni adjustment for multiple comparison) of the differences in RT for each emotional condition revealed that trials which commenced with negative emotional stimuli had the lowest RT in comparison to those which commenced with a positive (*p* < 0.001) or neutral (*p* = 0.02) stimuli (Figure 2A). Furthermore, there was also a significant difference in RT between trials which commenced with positive stimuli and those with neutral stimuli (*p* = 0.03). We also used another set of visual stimuli (NAPS image system) in another participant cohort (Supplementary Material Control Study 2). The results with the new image set replicated and confirmed that exposure to the negative stimuli enhanced performance, as indexed by the lowest RT and higher accuracy in comparison to positive and neutral stimuli exposure (Supplementary Figure S3).

**FIGURE 3:**
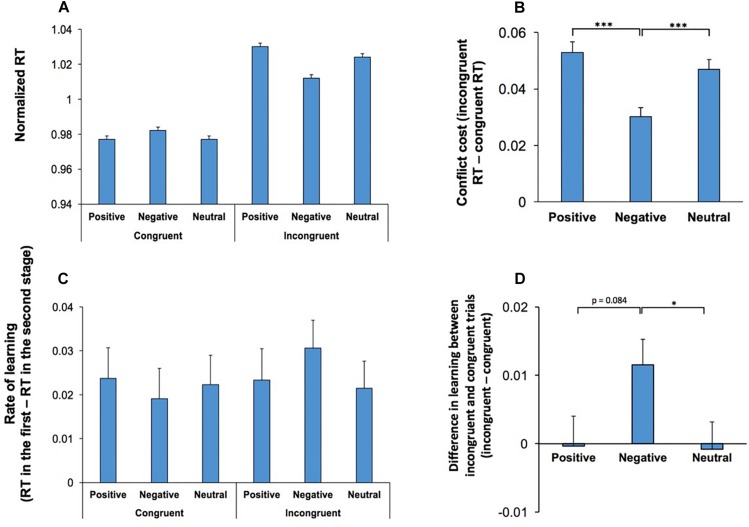
Behavioral effects of conflict were modulated by the emotional content of visual stimuli. **(A)** RT in correct trials are shown for congruent and incongruent trials for each emotional category. The effects of emotional stimuli was dependent on the conflict level. Incongruent trials, which commenced by negative stimuli had a decreased RT, while in congruent trials this effect was not observed. **(B)** The difference in RT between congruent and incongruent trials, conflict cost, is shown for each emotional category. Negative stimuli attenuated the magnitude of conflict cost. **(C)** Rate of learning (the difference in RT between the first to the second stage of testings in the same daily session) is shown for congruent and incongruent trials for each emotional category. Within session learning was dependent on the emotional valence and the conflict level. **(D)** Difference in learning between incongruent and congruent trials is shown (incongruent - congruent) for different emotional conditions. Learning decreased the conflict cost (difference between incongruent and congruent trials) in negative emotional conditions. ^*^represents *p* < 0.05, ^∗∗∗^represents *p* < 0.001.

There was also a significant interaction between Conflict and Emotion, *F*(2,108) = 26.37; *p* < 0.001, $\eta_{p}^{2}$ = 0.33, indicating that the effect of emotional stimuli was dependent on the level of conflict. RT was shorter in incongruent trials which contained negative stimuli however, such a decline in RT was not observed in congruent trials (Figure 3A). Figure 3B shows the difference in RT between congruent and incongruent trials (conflict cost for each emotional category). Pairwise comparisons (two-tailed *t* test with Bonferroni adjustment for multiple comparison) of the differences in conflict cost for each emotional condition revealed that negative stimuli attenuated the magnitude of conflict cost in comparison to positive (*p* < 0.001) and neutral (*p* < 0.001) stimuli.

The main effect of Music was not significant, *F*(2,108) = 1.93; *p* = 0.15, indicating that music did not influence RT. There was a significant interaction between Conflict, Emotion and Practice, *F*(2,108) = 3.26; *p* = 0.04, $\eta_{p}^{2}$ = 0.06, indicating that the rate of within-session learning was heightened in incongruent trials commencing with an emotionally negative stimulus (Figure 3C). To further assess this 3-way interaction, we calculated the difference in learning between congruent and incongruent trials for each emotional condition (Figure 3D). The learning related decline in conflict cost was significantly higher in negative condition when it was compared with neutral emotional condition (two-tailed *t* test with Bonferroni adjustment for multiple comparison *p* = 0.04). The difference between negative and positive conditions was not significant (*p* = 0.08). There were no other significant main effects, nor significant interactions.

The multi-factor ANOVA [Conflict × Music × Practice × Emotion] was also applied to the percentage of correct responses. The main effect of Conflict was significant, *F*(1,54) = 83.84; *p* < 0.001, $\eta_{p}^{2}$ = 0.61, indicating that accuracy was lower in incongruent trials. The main effect of Emotion was also significant, *F*(2,108) = 10.26; *p* < 0.001, $\eta_{p}^{2}$ = 0.16, indicating that the emotional content of the visual stimulus influenced accuracy (Figure 2B). Pairwise comparison of the differences in accuracy for each emotional condition (two-tailed *t* test with Bonferroni adjustment for multiple comparison) revealed that trials which commenced with negative visual stimuli had the highest accuracy in comparison to those which commenced with positive (*p* < 0.001) or neutral (*p* < 0.001) stimuli. The main effect of Practice, *F*(1,54) = 9.77; *p* = 0.003, $\eta_{p}^{2}$ = 0.15, was also significant. There were no other significant main effects, nor significant interactions. These findings indicate that the presentation of negative emotional stimuli at the beginning of a trial significantly improved the resolution of conflict between behavioral rules.

### Conflict Adaptation Was Influenced by Emotional Content of Visual Stimuli

**FIGURE 4:**
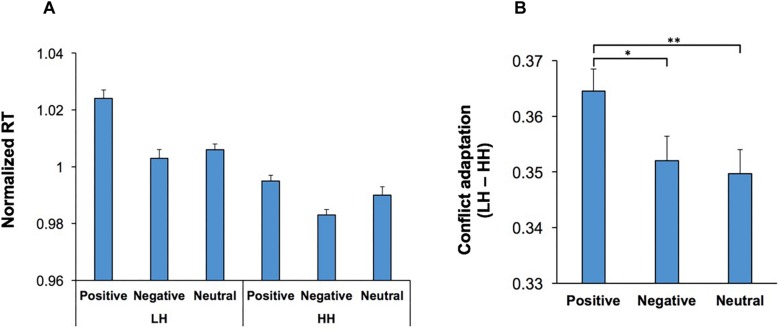
Conflict adaptation was modulated by the emotional content of visual stimuli. **(A)** The RT for incongruent trials that are immediately preceded by another incongruent trial (HH sequence), and those incongruent trials that are immediately preceded by a congruent trial (LH sequence) is shown for each emotional category. In both LH and HH sequences, trials which contained a negative start cue had lower RT than those which contained a positive start cue. **(B)** The magnitude of conflict adaptation (the difference in RT between LH and HH sequences) is shown for each emotional category. The magnitude of conflict adaptation was highest in trials which commenced with positive stimuli. ^*^represents *p* < 0.05, ^∗∗∗^represents *p* < 0.01.

The behavioral effects of conflict are not just limited to the current trial, and can also be observed in the subsequent trial, manifested as a behavioral improvement if the subject is faced with the same level of conflict again (conflict adaptation) (Gratton et al., 1992; Botvinick et al., 1999; Mayr et al., 2003; Mansouri et al., 2009). In the context of the WCST, this can be examined through contrasting incongruent trials that were immediately preceded by another incongruent trial (HH sequence) against those incongruent trials that were immediately preceded by a congruent trial (LH sequence). We examined whether conflict adaptation was influenced by emotion-modulating factors such as music and visual stimuli. A multi-factor ANOVA [Conflict Adaptation (HH/LH sequences, within-subject factor) × Music × Practice × Emotion] was applied to RT in the second trial in both HH and LH sequences. The main effect of Conflict Adaptation was significant, *F*(1,54) = 80.54; *p* < 0.001, $\eta_{p}^{2}$ = 0.60, and manifested as a lower RT in HH sequences than LH sequences (Figure 6A). The main effects of Practice, *F*(1,54) = 13.93; *p* = 0.01, $\eta_{p}^{2}$ = 0.21, and Emotion, *F*(2,108) = 17.27; *p* < 0.001, $\eta_{p}^{2}$ = 0.24, were also significant. Importantly, there was a significant interaction between Conflict Adaptation and Emotion, *F*(2,108) = 3.73; *p* = 0.03, $\eta_{p}^{2}$ = 0.07, indicating that the magnitude of conflict adaptation was differentially influenced by the emotional content of the visual stimuli (Figures 4A,B). For ease of comparison Figure 4A presents the normalized RT for both LH and HH sequences, while Figure 4B presents the magnitude of conflict adaptation (LH-HH). Pairwise comparisons (two-tailed *t* test with Bonferroni adjustment for multiple comparison) of the differences in conflict adaptation for each emotional condition revealed that the magnitude of conflict adaptation was increased in trials which commenced by positive stimuli in comparison to those which started with negative (*p* = 0.046) or neutral (*p* = 0.003) stimuli. However, this increase was primarily facilitated from the higher RT in LH sequences with positive stimuli (Figure 4A). There were no other significant main effects, nor significant interactions.

This ANOVA was also applied to the accuracy in the second trial in both HH and LH sequences. The main effect of Conflict Adaptation was significant, *F*(1,54) = 493.45; *p* < 0.001, $\eta_{p}^{2}$ = 0.90, indicating that accuracy was increased when the high conflict level was presented again (HH sequences). There were no other significant main effects, nor significant interactions.

### Conflict Processing in the WCST Influenced Emotional/Arousal State

We measured event-related EDA within two epochs: a post-feedback epoch [response selection onward (4 s)], to examine changes in arousal level after the participants became aware of their decision outcome, and a pre-feedback epoch [from the start cue to response feedback onset (2.4 s window)], to examine changes in arousal level before feedback.

**FIGURE 5:**
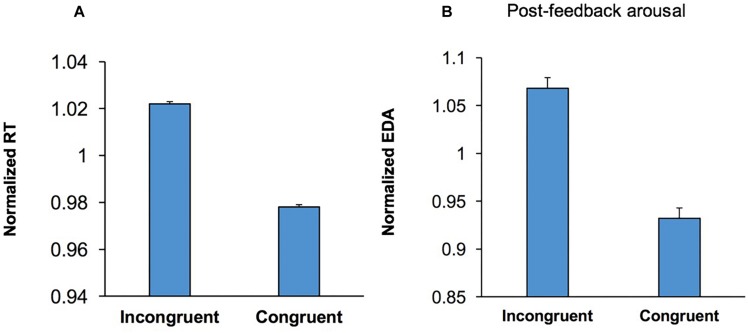
Conflict induced modulation in both behavior and emotional/arousal state. **(A)** The RT is shown for correct incongruent and congruent trials. RT was longer in incongruent trials than that congruent trials (conflict cost). **(B)** Post-feedback EDA is shown for correct incongruent and congruent trials. Post-feedback EDA was significantly higher following incongruent trials.

Participants’ RT was longer in incongruent trials than congruent trials (Figure 5A), indicating that conflict between behavioral options adversely affected performance in the WCST (conflict cost). To examine if processing conflict led to a shift in arousal level a multi-factor ANOVA [Conflict × Emotion × Music × Practice] was applied to post-feedback EDA in congruent and incongruent trials. The main effect of Conflict was significant, *F*(1,54) = 36.14; *p* < 0.001, $\eta_{p}^{2}$ = 0.40, and EDA was higher in incongruent trials. This indicates that conflict between behavioral options evoked a higher EDA and presumably a higher arousal level (Figure 5B). This suggests that following the behavioral effects of a higher level of conflict (Figure 5A), conflict-induced elevation in arousal level occurs as a sustained effect of conflict during the assessment of behavioral outcome (Figure 5B).

Although the emotional stimuli influenced behavior, no modulation of pre- or post-feedback arousal level was observed (*p* = 0.632 and *p* = 0.096. respectively). These indicate that the influence of emotional stimuli upon behavior was not necessarily mediated through changes in arousal level, but rather through a direct influence on executive control processes.

### Conflict Influenced Behavior and Arousal in the Following Trial

**FIGURE 6:**
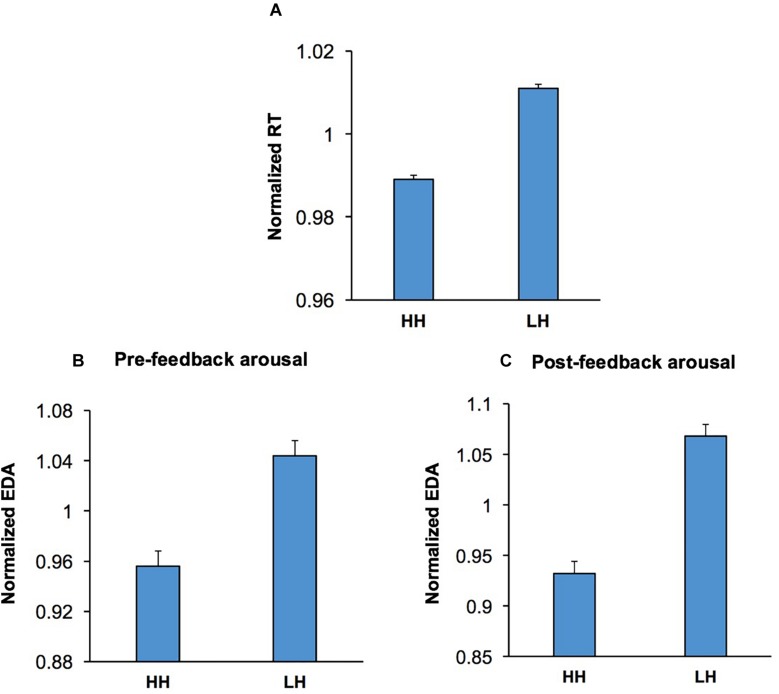
Conflict adaptation was accompanied via concurrent shifts in both pre- and post-feedback arousal. **(A)** The RT is shown for incongruent trials preceded by another incongruent trial (HH) and incongruent trials preceded by a congruent trial (LH). RT was shorter in HH sequences than LH sequences. **(B)** Pre-feedback EDA in the second trial in both HH and LH sequences are shown. The history of conflict level in the first trial in the sequences modulated pre-feedback EDA in the second, with incongruent trials preceded by another incongruent trial (HH) having lower pre-feedback EDA than those incongruent trials preceded by a congruent trial (LH sequences). **(C)** Post-feedback EDA in the second trial in both HH and LH sequences are shown. Similarly, to the pre-feedback epoch concomitant shifts in arousal accompanied conflict adaptation, with incongruent trials preceded by another incongruent trial (HH) having lower post-feedback EDA than those incongruent trials preceded by a congruent trial (LH sequences).

To examine whether conflict adaptation (Figure 6A) was also accompanied by shifts in emotional/arousal state, we applied a multi-factor ANOVA [Conflict Adaptation (HH/LH) × Emotion × Music × Practice] to the pre-feedback EDA in the second trial of HH and LH sequences. Pre-feedback EDA was selected for this analysis as the process of conflict adaptation must be triggered in the preceding trial and continue into the current trial to influence response selection. The ANOVA indicated that the main effect of Conflict Adaptation was highly significant, *F*(1,52) = 11.70; *p* = 0.001, $\eta_{p}^{2}$ = 0.20. This indicates that the process of conflict-induced behavioral adaptation is also reflected in changes in EDA and presumably in emotional/arousal state. In HH sequences, pre-feedback EDA was lower than that of LH sequences, implying that the process of conflict adaptation is accompanied by lower levels of arousal (Figure 6B). This suggests that those cognitive processes mediating the conflict-induced recruitment of executive control (Botvinick, 2007; Carter and van Veen, 2007; Mansouri et al., 2009, 2017b) also influence emotional/arousal state. There were no other significant main effects, nor significant interactions.

Furthermore, to assess if conflict adaptation is also accompanied by concurrent changes in post-feedback arousal level, we applied the multi-factor ANOVA [Conflict Adaptation × Emotion × Music × Practice] to the post-feedback EDA in the second trial in both HH and LH sequences. The main effect of Conflict Adaptation was significant, *F*(1,53) = 34.03; *p* < 0.001, $\eta_{p}^{2}$ = 0.39, indicating that conflict adaptation was also accompanied by post-feedback arousal shifts. The highest level of arousal was observed within LH trial sequences (Figure 6C). There were no other significant main effects, nor significant interactions.

These findings indicate that conflict induces alterations in both behavior and arousal level in the following trial. The improvement in behavioral performance seen as a shorter RT (Figure 6A) was accompanied by a lower arousal level in the periods before (Figure 6B) and after feedback (Figure 6C) in the second trial of HH sequences.

## Discussion

Our findings identified intriguing interactions between contextual factors with emotional content and conflict processing in the context of the WCST. We also assessed concomitant alterations in arousal level that reflected such behavioral alterations. We will discuss the significance of these findings in two aspects:

(1) Modulation of conflict resolution and conflict-induced behavioral adjustment by contextual emotional information.
(2) Alterations in arousal level in relation to the interaction between conflict processing and emotional information.

### Conflict Resolution Was Modulated by Contextual Emotional Information

Within the computerized WCST, the start cue was an image with emotional content of either positive, negative or neutral valence. We found that the emotional content of these stimuli induced significant changes in performance. RT was decreased (Figure 2A) and accuracy was enhanced (Figure 2B) in trials which commenced with negative stimuli. Executive functions and emotional regulation are intrinsically linked in goal-directed behavior (Bock, 2010; Garcia et al., 2011), with the somatic marker hypothesis proposing that alterations in emotional and arousal state may influence decision making (Bechara et al., 1999, 2005). In line with this hypothesis, our findings indicate that a brief exposure to emotional stimuli at the start of a trial, induced changes in emotional state and consequently influenced conflict resolution. Negative emotional stimuli distinctly enhanced performance, complimentary to the proposal that negative stimuli may be more salient than equivalent positive stimuli (McKenna and Sharma, 1995; Tipples and Sharma, 2000), due to evolutionary mechanisms which prioritize threat responses within the environment (Eysenck, 1997). In the context of an emotional Stroop task (McKenna and Sharma, 1995), performance was impaired in trials which contained negative stimuli. Albeit in the opposing direction, this result is compatible with our findings. Within their task, emotional stimuli were presented at response selection, whereas within our task the emotional stimuli were presented at the start of the trial. Therefore, in their study the heightened saliency of negative stimuli might have engaged attentional resources at the point of response selection and consequently disrupted performance. Whereas, within our study the heightened saliency of the negative stimuli presented at the start of the trial may heighten attention and executive control processes, consequently improving behavioral performance, as observed (Figures 2A,B). These findings indicate that the higher saliency of negative stimuli may modulate behavior across various tasks via augmenting attention, having the capacity to either heighten or impair performance dependent on when the stimuli is presented.

In our study, emotional content of visual stimuli modulated behavior, but did not alter EDA in pre- or post-feedback periods. Therefore, the effects of emotional stimuli were not necessarily mediated through alterations in trial-by-trial arousal level. Instead, emotionally salient stimuli might divert attention from extra-task events or inner mental world (attenuating mind wandering) toward the on-going task and therefore enhance upcoming task performance.

Conflict between available behavioral options impairs performance in numerous neuropsychological tests (Botvinick et al., 2001; Kerns et al., 2005; Carter and van Veen, 2007; Melcher et al., 2008; Mansouri et al., 2009, 2014, 2016a; Wessel et al., 2014). As demonstrated in this study, and past studies (Mansouri et al., 2009, 2016a), RT was longer in incongruent trials than congruent trials (Figure 5A). The behavioral effects of the emotional content of the start cue was dependent on the conflict level (Figure 3A) and appeared as a larger modulation in incongruent trials. This suggests that the influence of emotional state on performance is exaggerated when there is more demand for executive control of behavior.

We found significant practice-related learning within each testing session, which appeared as an improved ability in resolving conflict. Interestingly, this practice-related learning was also modulated by emotional state and was dependent on the conflict level (Figure 3C). Negative stimuli enhanced practice-related learning in incongruent trials, but had an opposite effect in congruent trials (Figure 3C). This suggests that within-session learning depends on an interaction between various contextual factors that influence executive and emotional regulation in the context of goal-directed behavior.

Our results do not fit within the emotional priming model which stems from the postulation that conflict is innately aversive (Botvinick, 2007; Padmala et al., 2011), and sensitive to emotional state (van Steenbergen et al., 2010). This idea gained support from a study where conflict-induced behavioral adjustments were attenuated when paired with a reward, as the reward diminished the aversive assessment of the conflict (van Steenbergen et al., 2010). Furthermore, this model was elaborated upon (Padmala et al., 2011) suggesting that heightening the aversive assessment of conflict, through the pairing of conflicting behavioral choices and negative stimuli, may increase the magnitude of conflict-induced behavioral adaptations (Padmala et al., 2011). The emotional priming model predicts that negative stimuli would heighten the aversive nature of the conflict, increasing conflict-induced behavioral adjustment manifested as an increased magnitude of conflict cost.

However, we found that negative stimuli instead attenuated the magnitude of conflict cost (Figure 3B), and therefore our results are in contrast to this model. Therefore, an alternative explanation could be that the influence of emotional content on conflict is mainly facilitated through the modulation of attention. As negative stimuli may be more salient than equivalent positive stimuli (McKenna and Sharma, 1995; Tipples and Sharma, 2000), the heightened level of attention induced by negative stimuli might enhance conflict resolution, and consequently attenuate conflict cost.

We found heightened practice-related learning in incongruent trials that commenced with negative emotional stimulus. It can be suggested that the heightened “attention capture” by negative stimuli promoted an increased rate of learning within incongruent trials (Figures 3C,D).

### Emotional Stimuli Influenced Executive Functions Without Changing Arousal Level

Although emotional visual stimuli influenced behavior, no modulation of pre- or post-feedback arousal was observed, indicating that the influence of emotional stimuli upon behavior was not necessarily mediated through changes in trial-by-trial arousal level. In our study, categorization of visual stimuli based on emotional content was done by independent raters and not by participants. They were also presented in a trial unique design. These were necessary to avoid the effects of repeated exposure to visual stimuli which could have evoked alterations in cognitive processes and arousal level due to familiarity and memory processing. We did not categorize visual stimuli based on the evoked arousal level because it could have introduced a bias in the outcome of our study and prevented assessing whether emotional stimuli induce their cognitive effects through changes in arousal level. We believe this was an important improvement (difference) in our task design, which allowed us to show “emotional stimuli affect executive functions without changing arousal level.” We may also assume that other features in visual stimuli such as complexity or low-level visual features, rather than the emotional content, could have led to behavioral modulations. We used a large set of stimuli, which were matched for their overall size and resolution and then categorized them by two independent assessors. Each participant was exposed to a large pool of stimuli in each category in a trial unique design in three consecutive weeks. Stimuli in each category included images with very different features. For example, the negative category included picture of a wound or image of a crying baby and therefore various colors, objects and complexity levels were included in each category. Therefore, it is highly unlikely that the behavioral effects were due to a particular feature or low-level visual information. The behavioral modulation was seen by negative stimuli and not by neutral or positive stimuli and therefore, attributing the differences to a difference in low-level visual information between the emotional categories and neutral images does not fit the findings.

Executive control and emotional regulation are intrinsically interrelated (Garcia et al., 2011), with imaging studies revealing a large overlap in brain areas which support executive control functions and those which regulate emotional state such as the amygdala, insula and prefrontal cortex (Craig, 2002; Bock, 2010; Mansouri et al., 2016b). As emotional stimuli modulated behavior without concurrent alterations in arousal level it can be proposed that this modulation occurred primarily through a direct influence on these executive control/emotional areas, rather than an alternative indirect influence facilitated via shifts in arousal level. It is important to acknowledge that shifts in arousal level accompanying conflict-induced behavioral adjustments were detected by our recording system. Therefore, the absence of a change in arousal level by the emotional stimuli was not due to a lack of sensitivity in the measurements. This framework diverges from a bulk of past research which has proposed that the influence of emotional content on behavior was primarily facilitated via changes in arousal level. Our findings instead indicate that emotional regulation is directly associated with cognitive flexibility and control of goal-directed behavior, and may be independent of changes in arousal level.

In contrast to the observed effects of emotional visual stimuli, which were dependent on valence, we did not find any significant modulation of performance depending on the music tempo. In Supplementary Material Control Study 1 we confirmed that participants could perceive differences between music conditions dependent on tempo, and therefore the absence of music effect was not because of an inability to distinguish differences in music characteristics. These findings suggest that in the context of the WCST emotional content of visual stimuli is more effective in modulating behavior than alterations in music tempo.

### Conflict-Induced Behavioral Adjustments Were Accompanied by Shifts in Arousal Level

We found that conflict-induced behavioral adjustment in the current trial (conflict cost) and in the subsequent trial (conflict adaptation), were accompanied by parallel changes in arousal level. In correct trials, post-feedback EDA was higher in incongruent than congruent trials (Figure 5B), indicating that the higher level of conflict present within incongruent trials evoked parallel shifts in both arousal and behavior (Figures 5A,B). This increase in arousal level in response to conflict was previously reported in other autonomic measures of arousal, such as pupil dilation (Brown et al., 1999; Gilzenrat et al., 2010; van Steenbergen and Band, 2013; Ebitz and Platt, 2015). Furthermore, we found that arousal was also influenced by conflict adaptation and appeared as a difference in EDA between the second trial of HH and LH sequences in both pre- and post-feedback epochs (Figures 6B,C). Pre-feedback arousal was lower in HH sequences than that of LH sequences. This shift in arousal level was dependent upon the conflict level in the preceding trial and presumably occurred concurrently as conflict-induced recruitment of executive control facilitated the conflict resolution in the second trial of HH sequences. Thus, it can be proposed that this arousal shift may be an autonomic manifestation of conflict-induced executive control adjustment. Moreover, within the post-feedback period arousal was still lower in HH sequences than that of LH sequences. These results are complimentary to a study which revealed concomitant shifts in pupil size, as an index of arousal, and conflict adaptation processes in the context of the Simon Task (van Steenbergen and Band, 2013), where the degree of pupil dilation positively correlated with the magnitude of conflict adaptation. An alternative hypothesis is that in the second trial of HH sequences, additional executive control processes are recruited and the system is in a better condition to resolve the conflict. This might decrease conflict-related uncertainty and therefore attenuate arousal levels in HH conditions.

## Limitations

In this study, participants were young adults within a limited age range (20–27 years old) and were all university students and therefore, it was a uniform cohort highly suitable for decreasing between-subject variabilities. However, generalizing our findings to other age ranges or the general population needs to be cautiously done. Future studies will be necessary to examine the interaction of emotional stimuli and conflict processing in children and elderly to verify whether the observed effects are age related. In addition, we used contemporary songs separated based on their tempo as background music. Future studies need to examine a broader range of music. The first set of visual stimuli used in this study were not rated by participants in terms of arousal or valence. However, in Control study 2, we used the second set of visual stimuli (NAPS images), which were rated based on arousal and valence by 204 people. Our findings with the first set were replicated and confirmed in Control study 2. Future studies may assess the correlation between subjective assessment of stimulus valence and arousal and the behavioral effects of emotional stimuli. In our study, the emotional stimuli were irrelevant to the task performance and therefore participants did not need to pay attention to them. It will be crucial to examine the interaction of emotional regulation and conflict processing when resolution of conflict depends on the processing of emotional information.

## Conclusion

We examined the effect of contextual factors with emotional content on conflict processing and arousal in the WCST. Our findings identified that brief exposure to emotional stimuli can modulate behavior, with behavior enhanced following negative stimuli. However, arousal was not modulated by emotional content. We have replicated and validated these findings in a control study (Supplementary Material Control Study 2) with a completely different set of images in another participant cohort. This additional study confirmed that the effects of emotional stimuli do not depend on a particular set or type of images and instead arises from emotional valence of visual images. We propose that the emotional modulation of behavior occurs primarily through a direct influence on highly interrelated brain areas which support conflict resolution and emotional regulation, rather than indirectly via changes in arousal level. Such an interaction might also be mediated through alterations in allocation of attention without altering arousal level. This novel framework diverges from a plethora of past research which postulated that the influence of emotion on behavior was primarily facilitated via changes in arousal level. Future research should more so consider the hypothesis that emotional regulation is directly associated with cognitive flexibility and control of goal-directed behavior, and may be independent of arousal level.

## Data Availability

The datasets for this manuscript are not publicly available because, other factors within the datasets are still being examined. However, datasets are available to editors and reviewers upon request. Requests to access the datasets should be directed to FM, farshad.mansouri@monash.edu.

## Ethics Statement

The approval was obtained from Monash University Human Research Ethics Committee. Written consent was obtained from all participants.

## Author Contributions

FM and DF designed the experiment, performed the analyses and wrote the manuscript. DF and RS collected the data. MR contributed to the manuscript preparation. All authors read and approved the final manuscript.

## Conflict of Interest Statement

The authors declare that the research was conducted in the absence of any commercial or financial relationships that could be construed as a potential conflict of interest.
