# Supplementary material for: Negative Emotional Stimuli Enhance Conflict Resolution Without Altering Arousal
Source: Front Hum Neurosci. 2019 Aug 13;13:282. doi: 10.3389/fnhum.2019.00282 (PMC6700260; doi:10.3389/fnhum.2019.00282)
Supplement: Supplementary file 1 [file Data_Sheet_1.PDF]

Supplementary Material

Control study 1: Examining the validity of the selected music conditions

Music conditions

The songs selected for inclusion were originally selected as a collection of pop songs used in a previous study (Mansouri et al., 2016). The lyrical music was then sorted via software into the high and low tempo conditions. Therefore, there was no bias in selecting and assigning the music into the relevant tempo category. To ensure the validity of the selected music conditions, Control study 1 was conducted (Mansouri et al., 2017) to systematically assess if other aspects of the music, such as 'Preference', 'Familiarity', 'Lyrics', 'Novelty' and 'Subjective mood' differed between the high and low tempo music conditions.

This control study was conducted in 14 participants (8 female) who were in the same age and education range as the participants of the main study. These participants were selected via a convenience sample and were paid for their time. Each participant listened to the same 28 songs (14 of each high and low tempo), which were used in the main study and were asked to rank them on 'Preference' (scale of 1-5), 'Familiarity' (scale of 1-5), interest in 'Lyrics' (no/neutral/yes), perceived 'Novelty' (old/neutral/new) and 'Subjective mood' (sad/neutral/happy) for each song. The songs played were intermingled and randomized from both tempo categories. Participants were blinded to the classification of the songs and completed no other tasks during the assessment of the songs. This subjective assessment could not be conducted during the completion of the main task as participants were performing the complex cognitive task, nor after, as the participants' memory of the ~25 songs played would not be reliable.

All 14 participants completed an assessment of all 28 songs. For each assessed ranking aspect (e.g. 'Preference') we conducted separate Chi-Square test of independence to examine whether there was an association between these aspects and music tempo (High/Low). A contingency table was formed for each factor. The null hypothesis for this analysis was that music tempo is independent of the other ranking aspects.

There was no significant association was found between the music tempo and participants: 'Preference' ($\chi^2_{0.05, 4} = 2.15$; $p = 0.71$) (Figure S1a), 'Familiarity' ($\chi^2_{0.05, 4} = 1.16$; $p = 0.88$) (Figure S1b), 'Lyrics' ($\chi^2_{0.05, 2} = 4.27$; $p = 0.12$) (Figure S1c), nor 'Novelty' ($\chi^2_{0.05, 2} = 4.72$; $p = 0.09$) (Figure S1d). However, there was a significant association found between music tempo and participants 'Subjective mood' ($\chi^2_{0.05, 2} = 29.47$; $p < 0.001$) (Figure S1e). In which participants associated high and low tempo music with a perceived happy and sad mood, respectively. This is in line with previous studies which have shown that music tempo is associated with mood changes (Hunter et al., 2010;Fernandez-Sotos et al., 2016;Mansouri et al., 2017), highlighting that the participants paid attention to categorizing each song and the selected approach was sensitive enough to dissociate these subjective aspects induced by music.

Interestingly within the main study, in contrast to significant effects of emotional content of visual stimuli, alterations in background music tempo nor lyrics had no significant effect on behavioural measures or arousal level in the context of the WCST. This control study confirmed that participants had a different perception of music conditions dependent on tempo, and therefore the absence of music effect on behaviour within the main study was not because of an inability to distinguish any influence of music types.

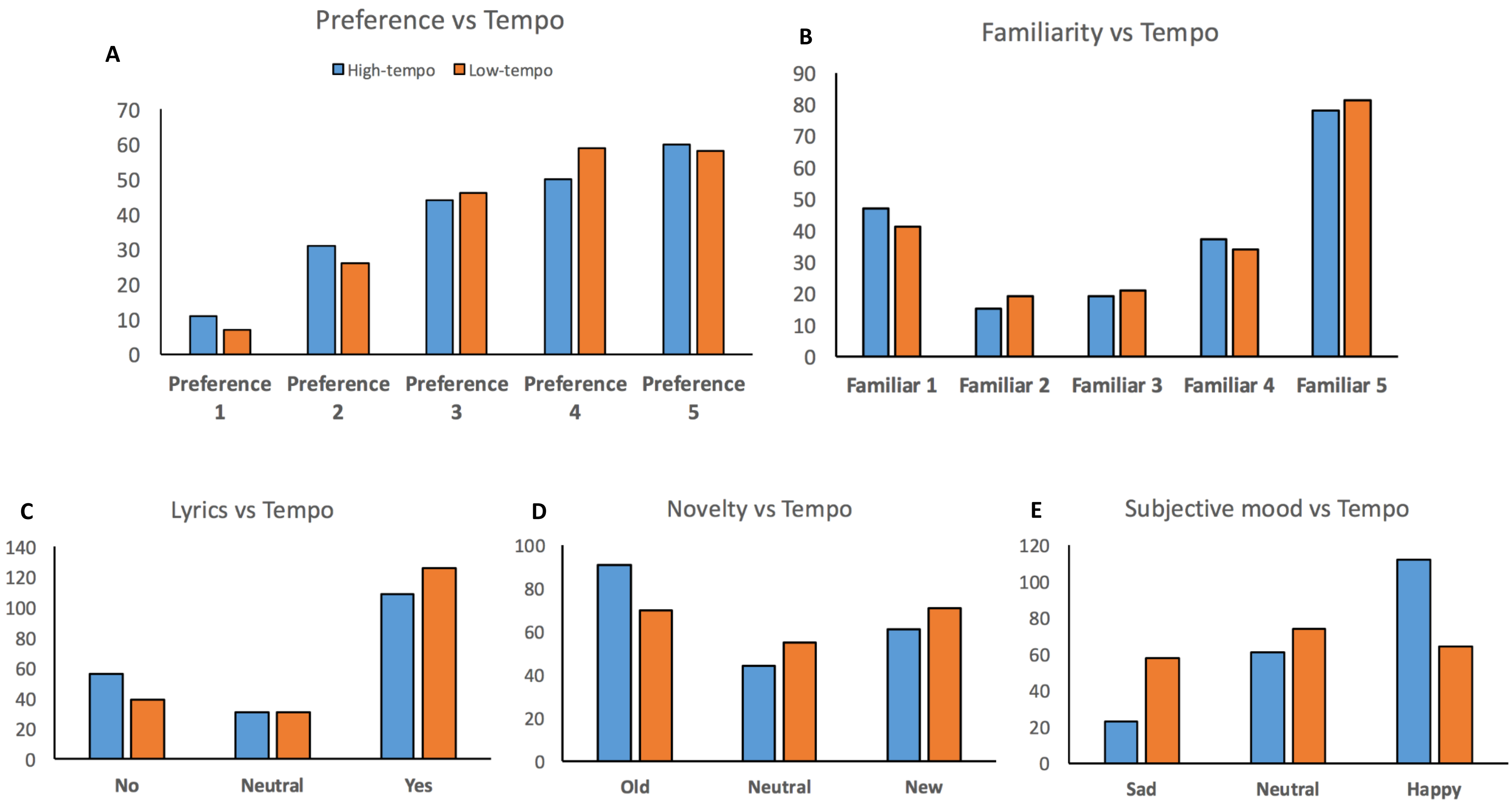

Supplementary figure 1. Subjective assessment of aspects with in high and low music categories. Subjective assessment induced via high and low music conditions in terms of 'Preference', 'Familiarity', 'Lyrics', 'Novelty' and 'Subjective mood'. Vertical axis shows the incidence rate of each condition.

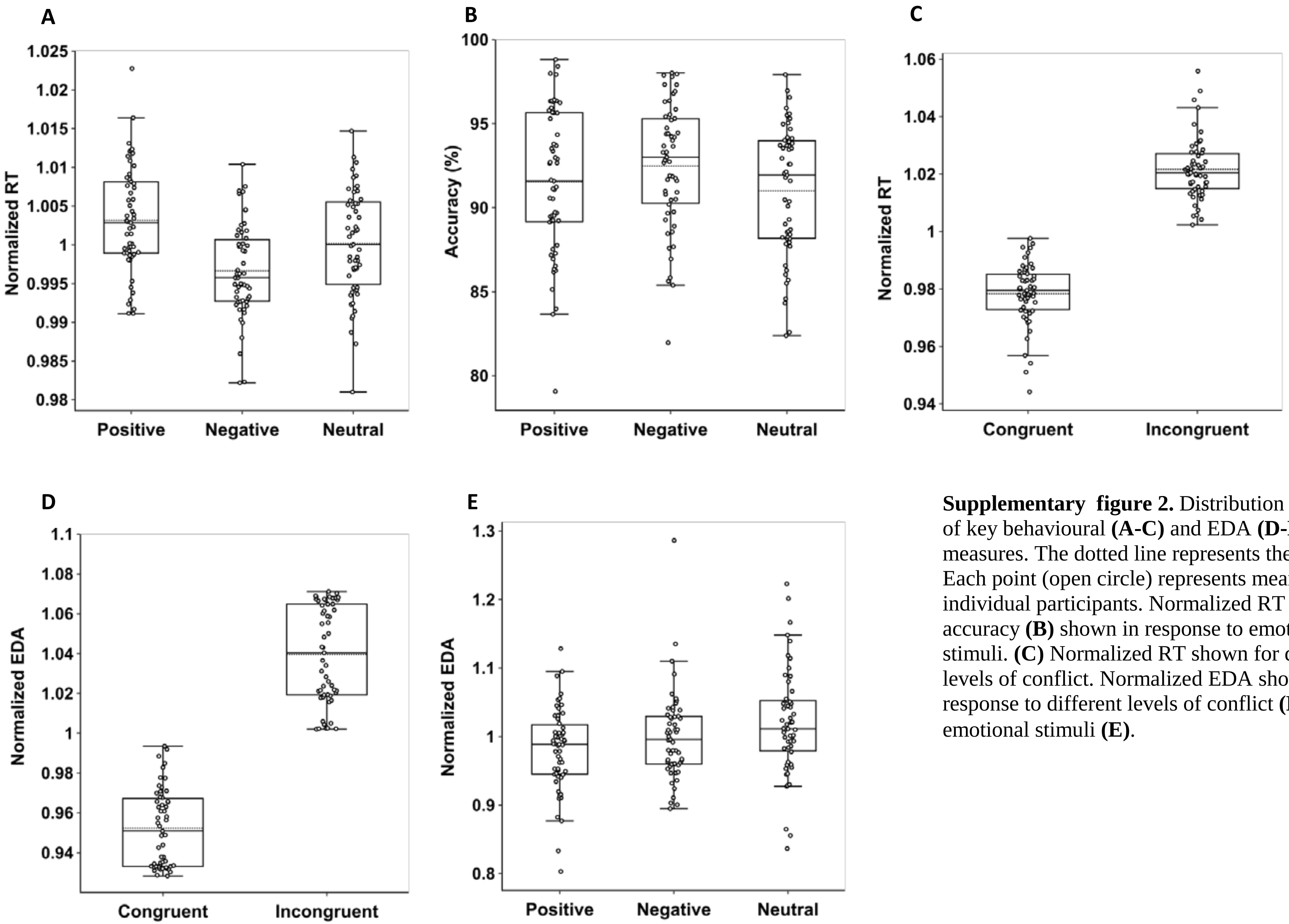

Supplementary figure 2. Distribution properties of key behavioural **(A-C)** and EDA **(D-E)** measures. The dotted line represents the mean. Each point (open circle) represents mean value for individual participants. Normalized RT **(A)** and accuracy **(B)** shown in response to emotion stimuli. **(C)** Normalized RT shown for different levels of conflict. Normalized EDA shown in response to different levels of conflict **(D)**, and emotional stimuli **(E)**.

Supplementary table 1. A selection of songs presented for each music category

Selection of presented songs					
Low		High		No-lyrics	
Song	Artist	Song	Artist	Song	Artist
Love Me Harder	Ariana Grande	Mr. Saxobeat	Alexandra Stan	Chord Left	Agnes Obel
Halo	Beyoncé	Pompeii (Audien Remix)	Bastille	Nocturnes, Op. 9	Chopin
If I Were a Boy	Beyoncé	Run the World (Girls)	Beyoncé	Drift	Daughter
Lanterns	Birds of Tokyo	How Deep Is Your Love	Calvin Harris & Disciples	Liebestraum	Franz Liszt
One Call Away	Charlie Puth	Safe and Sound	Capital Cities	Fascinating Rhythm	George Gershwin
Bloodstream	Ed Sheeran	Call Me Maybe	Carly Rae Jepson	Same Dream China	Gold Panda
Wide Awake	Katy Perry	3 Words	Cheryl Cole	Vanilla Minus	Gold Panda
Gold	Kiiara	Neon Lights	Demi Lovato	Into the Forest	Jennifer Thomas
Beneath You're Beautiful	Labrinth	Really Don't Care	Demi Lovato	Basique	Little People
Animals	Maroon 5	Hotline Bling	Drake	Fisticuffs at Dawn	Little People
One More Night	Maroon 5	Down	Jay Sean	Nuvole Bianche	Ludovico Einaudi
Just Give Me A Reason	Pink	Don't Be So Hard on Yourself	Jess Glynne	Symphony No. 35 in D Major	Mozart
1, 2, 3, 4	Plain White T's	What Do You Mean	Justin Bieber	iPlayYouListen	Odesza
Body on Me	Rita Ora	Moves Like Jagger	Maroon 5	Always This Late	Odesza
Stay with me	Sam Smith	Thank You	MKTO	Ballade pour Adeline	Richard Clayderman
Good for You	Selena Gomez	Counting Stars	One Republic	Matrimonio de amor	Richard Clayderman
Cheap Thrills	Sia	Don't Stop the Music	Rihanna	Almost Idyllic	Sleeping at Last
All My Friends	Snakehips	I Will Never Let You Down	Rita Ora	Arrival of The Birds	The Cinematic Orchestra
Hoops	The Rubens	Hello	Stafford Brothers	Ginza Samba	Vince Guaraldi
WILD	Troye Sivan	Clarity	Zedd	Charlie Brown Christmas	Vince Guaraldi

Supplementary Material

Control study 1: Examining perceived differences between music conditions dependent on tempo

Music conditions

The songs selected for inclusion were originally selected as a collection of pop songs used in a previous study (Mansouri et al., 2016). The lyrical music was then sorted via software into the high and low tempo conditions. Therefore, there was no bias in selecting and assigning the music into the relevant tempo category. To ensure the validity of the selected music conditions, Control study 1 was conducted (Mansouri et al., 2017) to systematically assess if other aspects of the music, such as 'Preference', 'Familiarity', 'Lyrics', 'Novelty' and 'Subjective mood' differed between the high and low tempo music conditions.

This control study was conducted in 14 participants (8 female) who were in the same age and education range as the participants of the main study. These participants were selected via a convenience sample and were paid for their time. Each participant listened to the same 28 songs (14 of each high and low tempo), which were used in the main study and were asked to rank them on 'Preference' (scale of 1-5), 'Familiarity' (scale of 1-5), interest in 'Lyrics' (no/neutral/yes), perceived 'Novelty' (old/neutral/new) and 'Subjective mood' (sad/neutral/happy) for each song. The songs played were intermingled and randomized from both tempo categories. Participants were blinded to the classification of the songs and completed no other tasks during the assessment of the songs. This subjective assessment could not be conducted during the completion of the main task as participants were performing the complex cognitive task, nor after, as the participants' memory of the ~25 songs played would not be reliable.

All 14 participants completed an assessment of all 28 songs. For each assessed ranking aspect (e.g. 'Preference') we conducted separate Chi-Square test of independence to examine whether there was an association between these aspects and music tempo (High/Low). A contingency table was formed for each factor. The null hypothesis for this analysis was that music tempo is independent of the other ranking aspects.

There was no significant association was found between the music tempo and participants: 'Preference' ($\chi^2_{0.05, 4} = 2.15$; $p = 0.71$) (Figure S1a), 'Familiarity' ($\chi^2_{0.05, 4} = 1.16$; $p = 0.88$) (Figure S1b), 'Lyrics' ($\chi^2_{0.05, 2} = 4.27$; $p = 0.12$) (Figure S1c), nor 'Novelty' ($\chi^2_{0.05, 2} = 4.72$; $p = 0.09$) (Figure S1d). However, there was a significant association found between music tempo and participants 'Subjective mood' ($\chi^2_{0.05, 2} = 29.47$; $p < 0.001$) (Figure S1e). In which participants associated high and low tempo music with a perceived happy and sad mood, respectively. This is in line with previous studies which have shown that music tempo is associated with mood changes (Hunter et al., 2010;Fernandez-Sotos et al., 2016;Mansouri et al., 2017), highlighting that the participants paid attention to categorizing each song and the selected approach was sensitive enough to dissociate these subjective aspects induced by music.

Interestingly within the main study, in contrast to significant effects of emotional content of visual stimuli, alterations in background music tempo nor lyrics had no significant effect on behavioural measures or arousal level in the context of the WCST. This control study confirmed that participants had a different perception of music conditions dependent on tempo, and therefore the absence of music effect on behaviour within the main study was not because of an inability to distinguish any influence of music types.

Control study 2: Replicating the behavioural effects of emotional stimuli with a new set of NAPS images.

To replicate and confirm our findings related to the cognitive effects of the visual stimuli with emotional content, we conducted a control study using images from the Nencki Affective Picture System (NAPS). NAPS is a large collection of multicolour, high-quality photographs classified into five categories (People, Faces, Animals, Objects, and Landscapes). This collection of images have numerous advantages over other commonly used pictures systems (Marchewka et al., 2014;Riegel et al., 2016). In NAPS, across the picture set, images are controlled in terms of 'dimension', 'luminance', 'entropy', and 'contrast' (Marchewka et al., 2014). In addition, rating of images for their affective-emotional content has been done by 204 participants according to separate criteria (Valence, Approach-avoidance and Subjective arousal). Rating in each criterion has been completed based on ranking from 1-9: for Valence [*very negative* (1) to *very positive* (9)], for Subjective arousal [from *relaxed* (1) to *aroused* (9)], and for Approach–avoidance [from *avoid* (1) to *approach* (9)]. In comparison to the other widely used picture system (International Affective Picture System, (Lang et al., 2008), the NAPS has a higher linear association between the valence and arousal dimensions across the picture set. Thus, with permission from the developers, we used images from the NAPS system as three separate categories of emotional images (Positive, Negative and Neutral) to assess replicability of our findings which were obtained with another set of visual images. The NAPS has been used and validated in different laboratories worldwide (Cudo et al., 2018;Mobius et al., 2018;Bovy et al., 2019;Ritchey et al., 2019).

Method

33 Monash University undergraduate students (19 female), with the same age range and education level as those in the main study, completed the computerized Wisconsin Card Sorting Test. All parameters (experimental and procedural) were the same as the main study however, the visual

stimuli (neutral and emotional) were selected from the NAPS. A subset of 360 images (same size of image pool as the main study) were randomly selected from the NAPS pool of images (120 for each emotional category: positive, negative, neutral). In NAPS, the images are rated in the scale of 1-9 by 204 people, based on the subjective impression of Affective valence. We assigned images to three separate categories (Neutral, Positive and Negative) based on each image's ranking for Affective valence (Marchewka et al., 2014). Images within a range of 1.00-2.50, 3.75-6.25 and 7.50-9.00 were used for the selection of Negative, Neutral and Positive image pool, respectively. These images were selected evenly across the five NAPS image categories (People, Faces, Animals, Objects, and Landscapes). Therefore, in our Control study, each one of image categories (positive, negative and neutral) included images of People, Faces, Animals, Objects, and Landscapes. The ranking criterion for selection of positive, negative and neutral images was the same for each image type (People, Faces, Animals, Objects, and Landscapes). The computerized version of the WCST used in this study included three rules: matching by colour, matching by shape and no-match rule where the subjects selected the test item that did not match the sample by either colour or shape. Participants completed this study over 2 sessions, one-week apart. In each weekly session, participants performed the cognitive task before and after a short period (10 minutes) of exercise or rest. The order of Exercise or Rest was counterbalanced. In Exercise session, participants exercised for 10 minutes on an exercise bike at 75-80% of maximum heartrate between the two testing stages. In Rest session, participants simply had a rest for 10 minutes between the testing sessions. Participants did not listen to music in either session, and only high conflict trials were presented. The Exercise factor is not relevant to the purpose of this Control study and did not interact with Emotion factor and therefore will not be reported here.

The main goal of this Control study (with a completely new set of emotional images) is to replicate and confirm two major findings that are reported in the main study:

1- The emotional content of visual stimuli influenced cognitive abilities in the executive control task (WCST) and appeared as a significant enhancement in performance following presentation of the negative stimuli.

2- Emotional content of visual stimuli did not change the participants' arousal level (assessed by measuring event-related electrodermal activity). This indicates that the emotional content of visual stimuli influenced cognitive functions without a concomitant change in arousal level.

Results

Response time (RT) and EDA were normalized, in the same way as the main study. For normalization, in each participant, the mean of each condition was calculated and then normalized by dividing each value by the grand average of all conditions.

Emotional content of emotional images significantly influenced participants' behaviour

We applied a multi-factor ANOVA [Rule (Color/Shape/No-Match, within-subject factor) × Emotion (visual stimuli with emotional content: positive/negative/neutral, within-subject factor) × Exercise (Exercise/Rest, within-subject factor) × Practice (pre-exercise/post-exercise, within-subject factor)] to the response time in correct trials. The main effect of Rule was significant ($F(2, 64) = 231.88$; $p < 0.001$, $\eta_p^2 = 0.88$) indicating that RT was modulated depending on the currently relevant rule. Importantly, there was a highly significant main effect of Emotion ($F(2, 64) = 13.95$; $p < 0.001$, $\eta_p^2 = 0.30$) indicating that the emotional content of the visual stimuli influenced RT. Pairwise comparisons (two-tailed t test with Bonferroni adjustment for multiple comparisons) of the differences in RT for each emotional condition revealed that trials which commenced with negative emotional stimuli had the lowest RT in comparison to those which commenced with a positive ($p < 0.001$) or neutral ($p = 0.001$) stimuli (Figure S3A).

The same multi-factor ANOVA [Rule × Emotion × Exercise × Practice] was also applied to the percentage of correct responses. The main effect of Rule was significant ($F(2, 64) = 185.90$; $p < 0.001$, $\eta_p^2 = 0.85$) indicating that accuracy was modulated by the matching rule. The main effect of Emotion was also significant ($F(2, 64) = 3.17$; $p = 0.049$ 1, $\eta_p^2 = 0.09$) indicating that the emotional content of the visual stimuli influenced accuracy, although the effect was much smaller than the Emotion effect on RT. Pairwise comparisons (two-tailed t test) were not significant after Bonferroni adjustment for multiple comparisons (negative vs positive ($p = 0.18$) and negative vs neutral ($p = 0.10$) (Figure S3B).

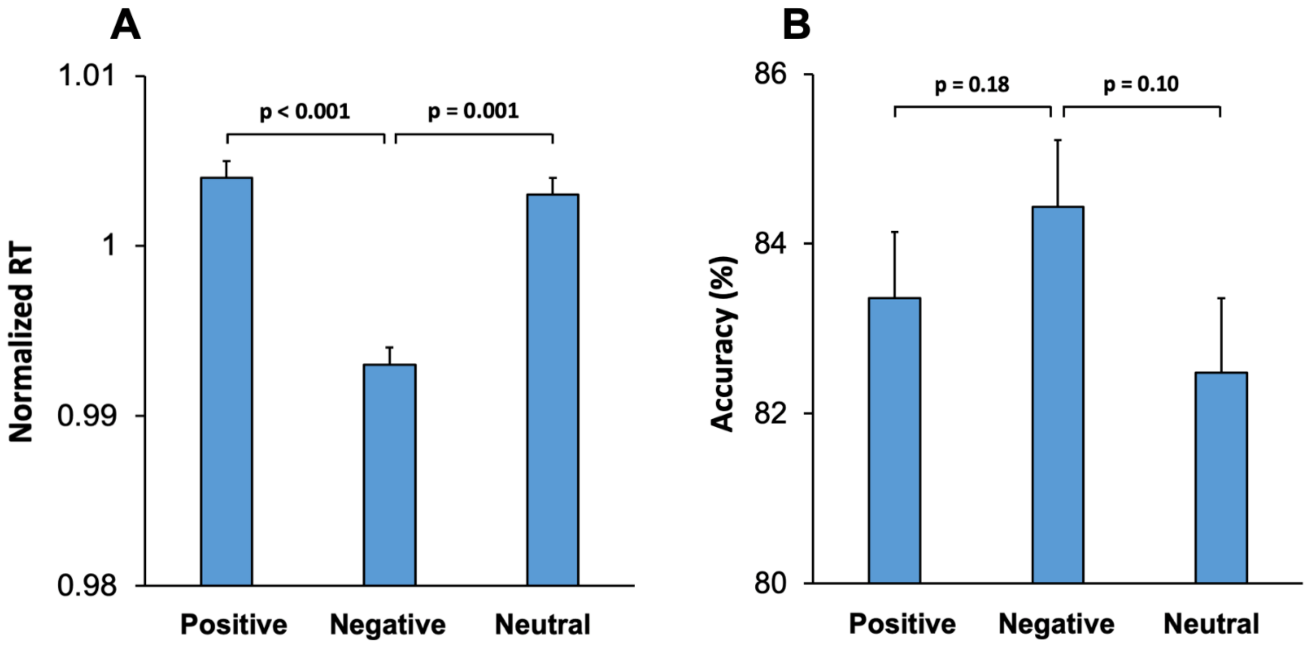

Supplementary figure 3. Emotional content of NAPS images influenced performance in the WCST. **(A)** Response time (RT) in correct trials are shown for each emotional category. RT was lowest in trials which contained a negative visual stimuli (shown as the start cue) **(B)** The percentage of correct trials are shown for each emotional category.

Emotional content of emotional images did not influence participants' arousal level

As in the main study, we also measured event-related electrodermal activity (EDA) as an index of arousal level, in two epochs: a post-feedback epoch (response selection onwards (4 sec)), to examine changes in arousal level after the participants became aware of their decision outcome, and a pre-feedback epoch (from the start cue to response feedback onset (2.4 sec window)), to examine changes in arousal level before feedback. To replicate and confirm the findings with the visual stimuli presented in the main study, we examined whether NAPS images affected participants' arousal level.

We applied a multi-factor ANOVA [Rule × Emotion (visual stimuli with emotional content: negative/ positive, neutral, within-subject factor) × Exercise × Practice] to post-feedback EDA in

correct trials. The main effect of Rule was significant ($F(2, 50) = 3.61$; $p = 0.03$, $\eta_p^2 = 0.13$). This indicates that, as was seen in the main study, participants' arousal was significantly modulated by the relevant rule. However, the main effect of Emotion was not significant ($F(1, 25) = 1.34$; $p = 0.26$, $\eta_p^2 = 0.05$). This indicates that the emotional content of NAPS images did not modulate arousal level. This also replicates our findings in the main study where we used another set of visual stimuli with emotional content. These findings indicate that ***emotional stimuli influenced performance but did not modulate the concomitant arousal level***. There were no other significant main effects nor interactions (all $p > 0.05$).

The same ANOVA structure was also applied to pre-feedback EDA in correct trials. Similarly, the main effect of Emotion was not significant ($F(1, 25) = 1.20$; $p = 0.28$, $\eta_p^2 = 0.05$) and there were no other significant main effects nor interactions (all $p > 0.05$).

Conclusion

Our Control study using NAPS images replicate and confirm two main findings of the main study:

(1) Visual stimuli with emotional content, particularly negative stimuli, modulated participants' performance in the cognitive task (Figure S3).

(2) The behavioural modulation by emotional stimuli occurred without concurrent changes in arousal level.

These findings replicate our findings and support our proposal that the behavioural effects of emotional stimuli on executive functions are not necessarily mediated through changes in arousal-emotional level and instead occur through direct modulation of executive control processes (Please see the Discussion section in the main text).

References

Bovy, L., Mobius, M., Dresler, M., Fernandez, G., Sanfey, A., Becker, E., and Tendolkar, I. (2019). Combining attentional bias modification with dorsolateral prefrontal rTMS does not attenuate maladaptive attentional processing. *Sci Rep* 9**,** 1168.

Cudo, A., Francuz, P., Augustynowicz, P., and Strozak, P. (2018). The Effects of Arousal and Approach Motivated Positive Affect on Cognitive Control. An ERP Study. *Front Hum Neurosci* 12**,** 320.

Fernandez-Sotos, A., Fernandez-Caballero, A., and Latorre, J.M. (2016). Influence of Tempo and Rhythmic Unit in Musical Emotion Regulation. *Front Comput Neurosci* 10**,** 80.

Hunter, P.G., Schellenberg, E.G., and Schimmack, U. (2010). Feelings and perceptions of happiness and sadness induced by music: Similarities, Differences, and Mixed Emotions. *Psychology of Aesthetics, Creativity, and the Arts* 4**,** 47-56.

Lang, J.P., Bradley, M.M., and Cuthbert, B. (2008). *International Affective Picture System (IAPS): Affective Ratings of Pictures and Instruction Manual (Rep. No. A-8).*

Mansouri, F.A., Acevedo, N., Illipparampil, R., Fehring, D.J., Fitzgerald, P.B., and Jaberzadeh, S. (2017). Interactive effects of music and brain stimulation in modulating executive functions. *Nature scientific reports* in press.

Mansouri, F.A., Fehring, D.J., Gaillard, A., Jaberzadeh, S., and Parkington, H. (2016). Sex dependency of inhibitory control functions. *Biol Sex Differ* 7**,** 11.

Marchewka, A., Zurawski, L., Jednorog, K., and Grabowska, A. (2014). The Nencki Affective Picture System (NAPS): introduction to a novel, standardized, wide-range, high-quality, realistic picture database. *Behav Res Methods* 46**,** 596-610.

Mobius, M., Ferrari, G.R.A., Van Den Bergh, R., Becker, E.S., and Rinck, M. (2018). Eye-Tracking Based Attention Bias Modification (ET-ABM) Facilitates Disengagement from Negative Stimuli in Dysphoric Individuals. *Cognit Ther Res* 42**,** 408-420.

Riegel, M., Żurawski, Ł., Wierzba, M., Moslehi, A., Klocek, Ł., Horvat, M., Grabowska, A., Michałowski, J., Jednoróg, K., and Marchewka, A. (2016). Characterization of the Nencki Affective Picture System by discrete emotional categories (NAPS BE). *Behavior research methods* 48**,** 600-612.

Ritchey, M., Wang, S.F., Yonelinas, A.P., and Ranganath, C. (2019). Dissociable medial temporal pathways for encoding emotional item and context information. *Neuropsychologia* 124**,** 66-78.
